# Timing of invasive mechanical ventilation and death in critically ill adults with COVID-19: A multicenter cohort study

**DOI:** 10.1371/journal.pone.0285748

**Published:** 2023-06-28

**Authors:** Adam Green, Jean-Sebastien Rachoin, Christa Schorr, Phil Dellinger, Jonathan D. Casey, Isabel Park, Shruti Gupta, Rebecca M. Baron, Shahzad Shaefi, Krystal Hunter, David E. Leaf, for the STOP-COVID Investigators

**Affiliations:** 1 Cooper University Health Care and Cooper Medical School of Rowan University, Camden, NJ, United States of America; 2 Division of Allergy, Pulmonary and Critical Care Medicine, Vanderbilt University Medical Center, Nashville, TN, United States of America; 3 Division of Renal Medicine, Brigham and Women’s Hospital, Boston, MA, United States of America; 4 Division of Pulmonary and Critical Care Medicine, Brigham and Women’s Hospital, Boston, MA, United States of America; 5 Department of Anesthesia, Critical Care and Pain Medicine, Beth Israel Deaconess Medical Center, Harvard Medical School, Boston, MA, United States of America; Universidad de La Sabana, COLOMBIA

## Abstract

**Purpose:**

To investigate if the timing of initiation of invasive mechanical ventilation (IMV) for critically ill patients with COVID-19 is associated with mortality.

**Materials and methods:**

The data for this study were derived from a multicenter cohort study of critically ill adults with COVID-19 admitted to ICUs at 68 hospitals across the US from March 1 to July 1, 2020. We examined the association between early (ICU days 1–2) versus late (ICU days 3–7) initiation of IMV and time-to-death. Patients were followed until the first of hospital discharge, death, or 90 days. We adjusted for confounding using a multivariable Cox model.

**Results:**

Among the 1879 patients included in this analysis (1199 male [63.8%]; median age, 63 [IQR, 53–72] years), 1526 (81.2%) initiated IMV early and 353 (18.8%) initiated IMV late. A total of 644 of the 1526 patients (42.2%) in the early IMV group died, and 180 of the 353 (51.0%) in the late IMV group died (adjusted HR 0.77 [95% CI, 0.65–0.93]).

**Conclusions:**

In critically ill adults with respiratory failure from COVID-19, early compared to late initiation of IMV is associated with reduced mortality.

## Introduction

Critically ill patients with coronavirus disease 2019 (COVID-19) who receive invasive mechanical ventilation (IMV) have in-hospital mortality rates as high as 40–50% [1, 2]. Despite the development of anti-inflammatory [3], immunomodulatory [4], and antiviral agents [5] to treat severe illness from COVID-19, mortality in this patient population remains extremely high. Additional therapeutic strategies are therefore urgently needed.

The timing of endotracheal intubation in critically ill patients with respiratory failure from COVID-19 has been debated since the beginning of the pandemic. Proponents of early intubation often cite the risk of patient self-inflicted lung injury (P-SILI) [6, 7]. Under this framework, vigorous spontaneous breathing in a non-intubated patient contributes to acute lung injury due to large tidal volumes (strain) and transpulmonary pressure fluctuations (stress) [8, 9]. Those who oppose an early intubation approach cite the high mortality in patients receiving IMV, as well as the unproven concepts behind P-SILI [10].

Conflicting evidence exists regarding timing of intubation and associated outcomes. In a retrospective cohort study of 755 adult patients with severe COVID-19, earlier intubation following hospital admission was associated with improved survival [11]. In contrast, a systematic review of cohort studies found no association between timing of intubation and mortality [12]. However, without comprehensive adjustment for disease severity, including changes in clinical trajectory, these results are challenging to interpret.

We sought to bring further clarity to this question by leveraging data from a large, nationally-representative, multicenter cohort study of critically ill patients with COVID-19. We specifically investigated the association between early versus late initiation of IMV and survival.

## Study design and methods

### Study design and oversight

We used data from the Study of the Treatment and Outcomes in Critically ill Patients with COVID-19 (STOP-COVID), a multicenter cohort study that enrolled consecutive adults with laboratory-confirmed COVID-19 admitted to participating ICUs at 68 hospitals across the United States. Study personnel at each site collected data by detailed chart review and used a standardized electronic case report form (Research Electronic Data Capture [REDCap]) to enter data into a secure online database. All data were validated through a series of automated and manual checks. Additional details on STOP-COVID, including a complete list of variables collected and a list of participating sites, are reported elsewhere [1]. STOP-COVID was approved with a waiver of informed consent by the Institutional Review Board (IRB) at each participating site (protocol number 2007000003 for the Mass General Brigham IRB).

### Eligibility criteria

We included adult patients (≥18 years old) with laboratory-confirmed COVID-19 admitted to an ICU between March 1 and July 1, 2020. In order to meet eligibility criteria for the parent study, patients had to be admitted to the ICU for illness directly attributable to COVID-19. For the current study, we excluded patients who did not initiate IMV on ICU days 1–7, those who were transferred from another hospital, and those who were hospitalized for one week or longer prior to ICU admission (the latter two exclusion criteria were chosen to minimize the potential misclassification of patients into “early” versus “late” initiation of IMV groups). We also excluded patients with any of the following clinical features on ICU days 1 or 2: receipt of extracorporeal membrane oxygenation (ECMO); partial pressure of arterial oxygen over the fraction of inspired oxygen (PaO_2_:FiO_2_) <100 mm Hg; arterial pH <7.0; lactate >10 mmol/L; receipt of 3 or more vasopressors/inotropes; or cardiac arrest. These criteria were chosen to parallel those that would be used in a hypothetical target trial of early versus late initiation of IMV [13].

### Primary exposure

We categorized patients according to whether they initiated IMV “early” versus “late”. Early initiation of IMV was defined as within the first 2 days of ICU admission, similar to criteria used in several prior studies [14, 15]. Late initiation of IMV was defined as occurring on ICU days 3–7. We limited the treatment exposure to 7 days following ICU admission in the late initiation group to minimize heterogeneity between patients and allow more follow-up time.

### Follow-up and outcomes

The primary outcome was time to in-hospital death, censored at the first of hospital discharge or 90 days following initiation of IMV.

### Statistical analysis

#### Overview

The primary analysis compared time to death among patients who initiated IMV early versus late. Hazard ratios (HRs) and 95% confidence intervals (CIs) were estimated using a multivariable Cox model. To minimize the potential for immortal time bias, follow-up for each patient began on the day of IMV initiation. The following covariates were prespecified based on clinical knowledge [1], biologic plausibility, completeness of data, and parsimony: age; sex; race; body mass index (BMI); hypertension; diabetes mellitus; coronary artery disease; congestive heart failure (CHF); chronic lung disease; active malignancy; days from symptom onset to ICU admission; hospital size (number of pre-COVID-19 ICU beds); and severity-of-illness characteristics assessed on the day of IMV initiation (the renal, liver, and coagulation components of the Sequential Organ Failure Assessment [SOFA] score [16], the PaO_2_:FiO_2_ ratio, shock, concurrent therapies received [corticosteroids; tocilizumab; prone positioning; neuromuscular blockade], and inflammation). Concurrent therapies were included if they were administered on the day of IMV initiation or prior. Inflammation was defined using predefined thresholds for C-reactive protein (>100 mg/L), interleukin-6 (>80 pg/ml), and ferritin (>1,000 ng/mL). Additional details are provided in the **Supplemental Methods in S1 File**.

In addition to the time-to-death analyses described above, we also estimated the difference in the risk of 90-day mortality in the early versus late initiation of IMV groups using a multivariable logistic regression model. In this model, patients discharged prior to day 90 were assumed to still be alive at day 90.

#### Sensitivity and exploratory analyses

We conducted two prespecified sensitivity analyses, one post-hoc sensitivity analysis, and one post-hoc exploratory analysis. First, we kept discharged patients in the risk set until day 90, since Cox models assume non-informative censoring. Second, as an alternative approach to minimize immortal time bias, we repeated the primary analysis but excluded patients who died in the first 7 days of ICU admission. In this analysis, follow-up for each patient began 8 days after ICU admission. Third, we repeated the primary analysis and further adjusted for date of ICU admission (before vs. after the median date of ICU admission for the cohort, which was April 1, 2020), hospital geographic region (Northeast, South, Midwest, and West), and hospital type (main hospital vs. satellite/affiliate). We also conducted an exploratory analysis in which patients not intubated on ICU days 1–7 were included in the late IMV group. The inclusion and exclusion criteria for this exploratory analysis were similar to the primary analysis, except that patients who received no supplemental oxygen or only minimal supplemental oxygen (nasal cannula or simple face mask) on ICU admission were excluded to reduce confounding. For the exploratory analysis, severity-of-illness characteristics were assessed on ICU admission, which also served as the start of follow-up.

#### Subgroup analyses

We used similar methods as the primary analysis described above to assess the effect of early versus late initiation of IMV on time to death across the following prespecified subgroups: age (<60 vs. ≥60 years); sex; race; BMI (<40 vs. ≥40 kg/m^2^); chronic lung disease (composite of COPD or current or former smoker); time from symptom onset to ICU admission (≤3 vs. >3 days); inflammation on the day of IMV initiation or prior; corticosteroid use on the day of IMV initiation or prior; and hospital size (≥100, 50–99, or <50 pre-COVID ICU beds). We compared differences among subgroups by adding product (“interaction”) terms between the subgroup variable and the IMV group into the multivariable model. All comparisons are two tailed, with P<0.05 considered significant. Because of multiple comparisons, findings for subgroup analyses should be interpreted as exploratory.

#### Missing data

The renal, liver, and coagulation components of the SOFA score were categorized as “0” if missing [17–19]. Otherwise, missing data were not imputed. Rather, we created a separate missing category for each covariate that had missing data, since data may not have been missing at random. Further, the missingness of a variable could have clinical relevance (e.g., a healthier patient may not have certain physiologic or laboratory values assessed as frequently), which could affect treatment decisions.

#### Assessment of baseline characteristics

Continuous variables are expressed as median and interquartile range (IQR) and categorical variables are presented as count and percentage. Differences in baseline characteristics between early versus late initiators of IMV were analyzed with a *t* test or Mann–Whitney U test for continuous data and with a Chi-square or Fisher’s exact test for categorical data, as appropriate. All analyses were performed using SPSS 27 (IBM, Armonk, NY).

## Results

### Patient characteristics

**Fig 1:**
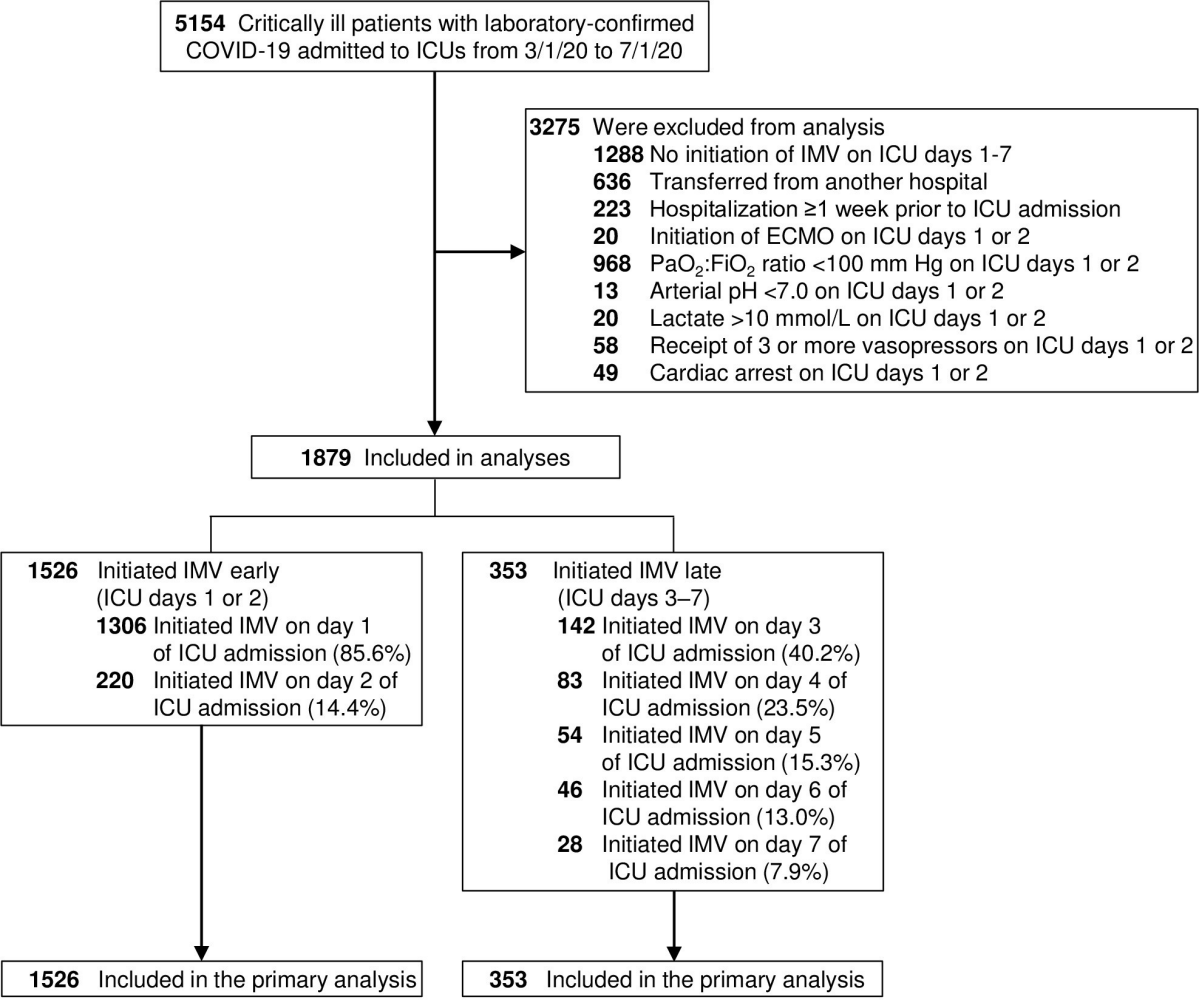
Study cohort and emulated trial flow. Abbreviations: ECMO, extracorporeal membrane oxygenation; IMV, invasive mechanical ventilation.

Among 5154 patients enrolled in STOP-COVID, 1879 (36.5%) were included in this analysis (**Fig 1**). The median age was 63 years (IQR, 53–72) and 1199 patients (63.8%) were male. A total of 1526 of the 1879 patients (81.2%) initiated IMV early, and 353 (18.8%) initiated IMV late. The characteristics of patients who initiated IMV early versus late are shown in **Table 1**. Patients who initiated IMV early had similar distributions of age, sex, race, BMI, most comorbidities, time from symptom onset to ICU admission, and shock and inflammation on the day of IMV initiation as compared to patients who initiated IMV late. Patients in the two groups were also similar with respect to the proportion admitted to larger versus smaller hospitals (according to number of ICU beds). However, patients who initiated IMV late were more likely to have CHF, chronic lung disease, moderate-to-severe hypoxemia (PaO_2_:FiO_2_ ratio <200 mm Hg), and a higher renal SOFA score on the day of IMV initiation compared to those who initiated IMV early. Patients who initiated IMV late were also more likely to have received corticosteroids, tocilizumab, prone positioning, and neuromuscular blockade (**Table 1**).

**Table 1:** Baseline characteristics.

	Treatment group		
Characteristic	Early initiation of IMV (n = 1526)	Late initiation of IMV (n = 353)	P Value
**Demographics**			
Age–median (IQR)	63 (53─72)	63 (53─73)	0.68
Age–no. (%)			
18–49	286 (18.7)	66 (18.7)	0.91
50–59	312 (20.4)	69 (19.5)	
60–69	460 (30.1)	103 (29.2)	
≥70	468 (30.7)	115 (32.6)	
Male sex–no. (%)	983 (64.4)	216 (61.2)	0.26
Race–no. (%)			
White	606 (39.7)	137 (38.8)	0.76
Non-White/Other/Unknown	920 (60.3)	216 (61.2)	
BMI (kg/m^2^)–median (IQR)	29.9 (26.1─34.6)	30.3 (26.4─36.7)	0.14
**Coexisting conditions–no. (%)**			
Hypertension	935 (61.3)	222 (62.9)	0.57
Diabetes mellitus	648 (42.5)	151 (42.8)	0.92
Coronary artery disease	187 (12.3)	56 (15.9)	0.07
Congestive heart failure	132 (8.7)	49 (13.9)	0.003
Chronic lung disease	471 (30.9)	146 (41.4)	<0.001
Active malignancy	61 (4.0)	19 (5.4)	0.25
**Days from symptom onset to ICU admission–no. (%)**			
≤3	346 (22.8)	95 (27.1)	0.23
>3	1173 (77.2)	256 (72.9)	
**Days from ICU admission to IMV initiation–median (IQR)**	1 (1–1)	4 (3–5)	<0.001
**Severity-of-illness–no. (%)** ^**a**^			
PaO_2_:FiO_2_, mm Hg^b^			
≥300	165 (13.5)	11 (3.8)	<0.001
200–299	318 (26.1)	30 (10.2)	
<200	735 (60.3)	252 (86.0)	
Shock^c^	166 (10.9)	48 (13.6)	0.15
Renal SOFA score^d^			
0	1033 (67.7)	236 (66.9)	0.007
1	177 (11.6)	28 (7.9)	
2	81 (5.3)	12 (3.4)	
3	111 (7.3)	31 (8.8)	
4	124 (8.1)	46 (13.0)	
Liver SOFA score^e^			
0	1386 (90.8)	320 (90.7)	0.13
1	105 (6.9)	19 (5.4)	
2–4	35 (2.3)	14 (4.0)	
Coagulation SOFA score^f^			
0	1215 (79.6)	298 (84.4)	0.10
1	235 (15.4)	44 (12.5)	
2–4	76 (5.0)	11 (3.1)	
Inflammation^g^			
Inflamed (≥1 elevated marker)	251 (22.3)	49 (21.2)	0.71
Non-inflamed (no elevated markers)	873 (77.7)	182 (78.8)	
**Treatments–no. (%)** ^**h**^			
Corticosteroids	218 (14.3)	103 (29.2)	<0.001
Tocilizumab	241 (15.8)	88 (24.9)	<0.001
Prone positioning	609 (39.9)	166 (47.0)	0.01
Neuromuscular blockade	596 (39.1)	163 (46.2)	0.01
**Number of ICU beds–no. (%)**			
<50	537 (35.2)	118 (33.4)	0.51
50–99	426 (27.9)	93 (26.3)	
≥100	563 (36.9)	142 (40.2)	

^a^Severity-of-illness characteristics were assessed on the day of IMV initiation.

^b^PaO_2_:FiO_2_ was only assessed in patients receiving IMV. If multiple PaO_2_ values were available on the same day, the lowest value was recorded, along with the corresponding FiO_2_.

^c^Defined as receipt of two or more vasoactive agents, including phenylephrine, epinephrine, norepinephrine, vasopressin, dopamine, dobutamine, and milrinone.

^d^Renal SOFA scores were calculated by considering the daily SCr, the daily UOP, receipt of RRT, and ESRD. Category 0, SCr <1.2 mg/dl and UOP ≥500 ml; category 1, SCr 1.2–1.9 mg/dl and UOP ≥500 ml; category 2, SCr 2.0–3.4 mg/dl and UOP ≥500 ml; category 3, SCr 3.5–4.9 mg/dl or UOP <500 ml; category 4, SCr ≥5 mg/dl, UOP <200 ml, receipt of RRT, or ESRD. Higher scores indicate more severe renal dysfunction.

^e^Liver SOFA scores were calculated by determining the daily bilirubin level. Category 0, bilirubin <1.2 mg/dl; category 1, bilirubin 1.2–1.9 mg/dl; category 2–4, bilirubin ≥2 mg/dl. Higher scores indicate more severe liver dysfunction. Categories 2, 3, and 4 were binned due to a low frequency of events in categories 3 and 4.

^f^Coagulation SOFA scores were calculated by determining the daily platelet level (per mm^3^). Category 0, platelet count ≥150; category 1, platelet count 100–149; category 2–4, platelet count <100. Higher scores indicate more severe dysfunction of the coagulation system. Categories 2, 3, and 4 were binned due to a low frequency of events in categories 3 and 4.

^g^Inflamation was defined as at least one of the following on the day of IMV initiation or prior: C-reactive protein >100 mg/L, interleukin-6 >80 pg/ml, or ferritin >1,000 ng/mL. Non-inflamed was defined as at least one value below the thresholds above, and no values that were above the thresholds. These thresholds above were selected based on prior studies [20–22].

^h^Refers to treatments received on the day of IMV initiation or prior.

Abbreviations: BMI, body mass index; ESRD, end stage renal disease; ICU, intensive care unit; IMV, invasive mechanical ventilation; PaO_2_:FiO_2_, ratio of the partial pressure of arterial oxygen over the fraction of inspired oxygen; RRT, renal replacement therapy; SCr, serum creatinine; SOFA, Sequential Organ Failure Assessment; UOP, urine output.

Data regarding BMI were missing for 51 patients (3.3%) in the early IMV group and 16 patients (4.5%) in the late group.

Data regarding active malignancy were missing for 2 patients (0.1%) in the early IMV group and in 0 patients in the late group.

Data regarding days from symptom onset to ICU admission were missing for 7 patients (0.5%) in the early IMV group and 2 patients (0.6%) in the late group.

Data regarding PaO_2_:FiO_2_ were missing for 308 patients (20.2%) in the early IMV group and 60 patients (17.0%) in the late group.

Data regarding inflammation were missing for 402 patients (26.3%) in the early IMV group and 122 patients (34.6%) in the late group.

All other data are complete.

### Mortality

**Fig 2:**
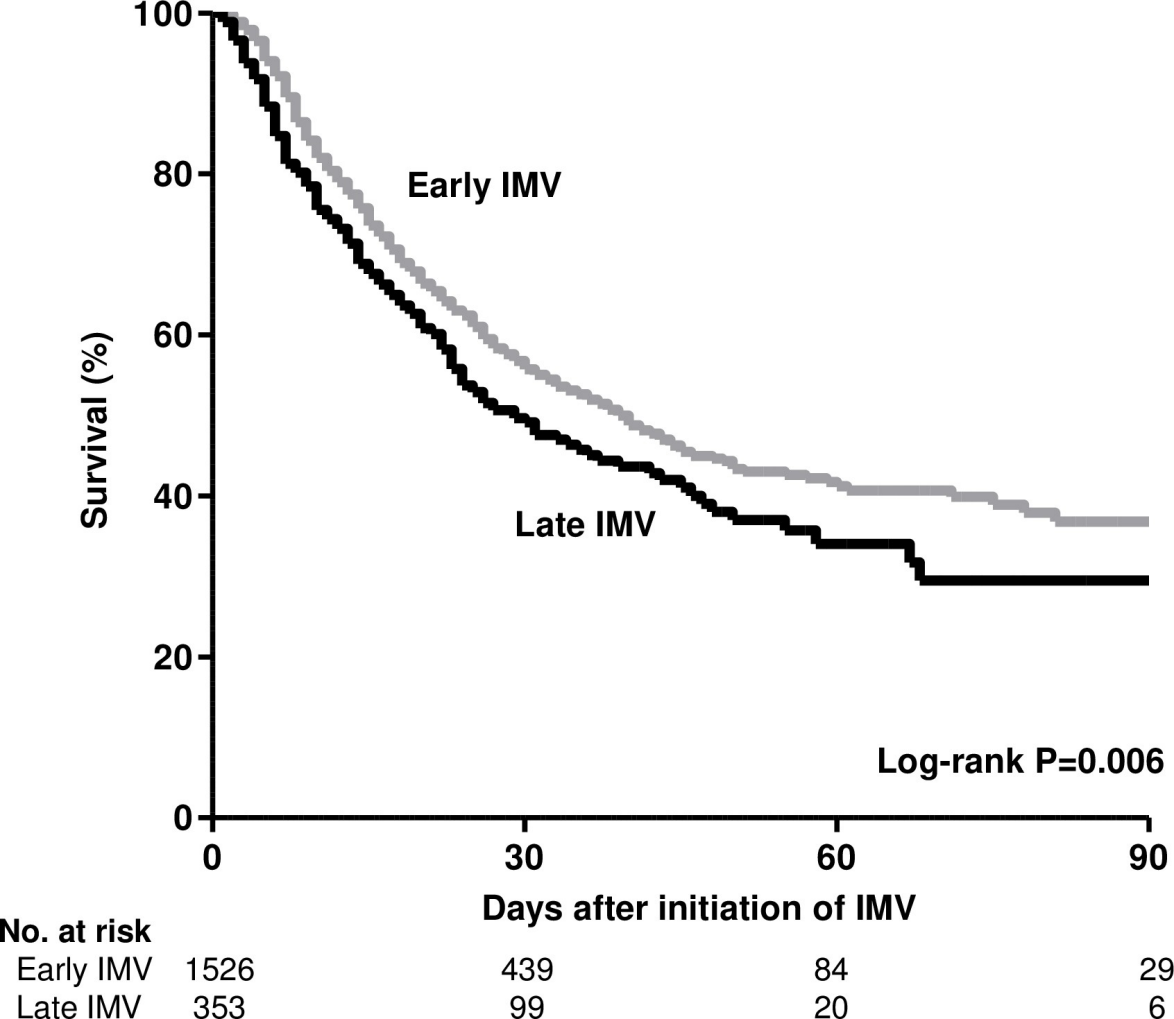
Mortality in early versus late initiation of IMV groups. Abbreviations: IMV, invasive mechanical ventilation.

The median follow-up for the patients who initiated IMV early and late was 18 (IQR, 10–31) and 19 (IQR, 9–30) days, respectively (overall, 18 [IQR, 10–31] days). A total of 824 patients (43.9%) died, 1035 (55.1%) were discharged alive, and 20 (1.1%) remained hospitalized at day 90. The 824 patients who died included 644 of the 1526 (42.2%) who initiated IMV early and 180 of the 353 (51.0%) who initiated IMV late (unadjusted HR, 0.80 [95% CI, 0.67–0.94]; **Fig 2**). Causes of death are shown in **S1 Table in S1 File**.

**Fig 3:**
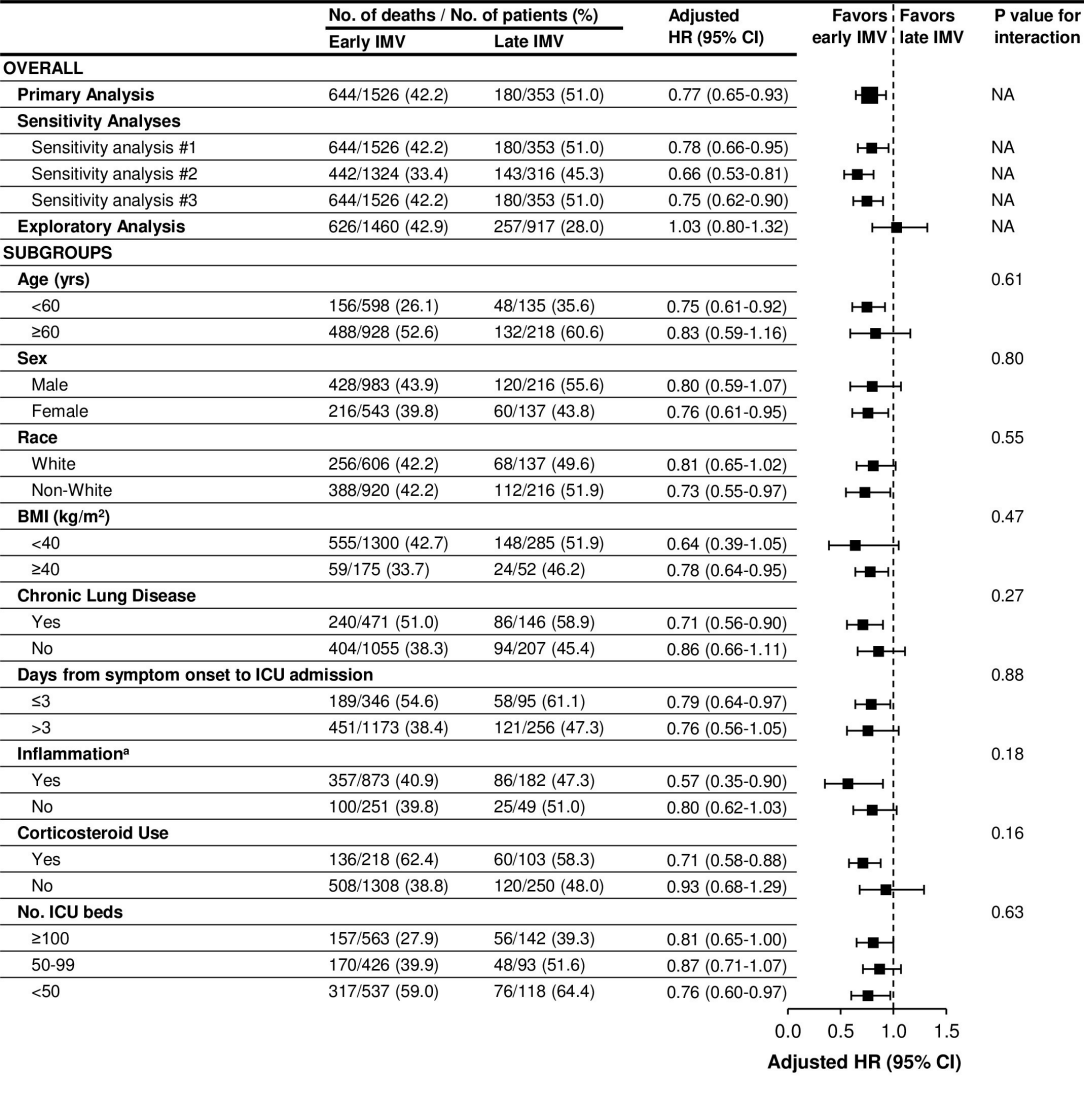
Primary and subgroup analyses examining mortality in early versus late initiation of IMV groups. The hazard ratios are adjusted for the following covariates: age; sex; race; body mass index; hypertension; diabetes mellitus; coronary artery disease; congestive heart failure; chronic lung disease; active malignancy; days from symptom onset to ICU admission; hospital size (number of pre-COVID-19 ICU beds); and severity-of-illness covariates assessed on the day of IMV initiation (the renal, liver, and coagulation components of the Sequential Organ Failure Assessment score [13], the PaO_2_:FiO_2_ ratio, shock, concurrent therapies received [corticosteroids; tocilizumab; prone positioning; neuromuscular blockade], and inflammation. Sensitivity analysis #1 kept discharged patients in the risk set until day 90. Sensitivity analysis #2 excluded patients who died in the first 7 days of ICU admission, with follow-up for each patient starting on day 8 after ICU admission. Sensitivity analysis #3 was further adjusted for date of ICU admission, hospital geographic region, and hospital type. The exploratory analysis included patients not intubated during ICU days 1–7 in the “late IMV” group. Abbreviations: BMI, body mass index; HR, hazard ratio; IMV, invasive mechanical ventilation. ^a^Inflamation was defined as at least one of the following on the day of IMV initiation or prior: C-reactive protein >100 mg/L, interleukin-6 >80 pg/ml, or ferritin >1,000 ng/mL. Non-inflamed was defined as at least one value below the thresholds above, and no values that were above the thresholds. These thresholds above were selected based on prior studies [20–22].

In the primary analysis, patients who initiated IMV early had a lower adjusted risk of death compared to patients who initiated IMV late (HR, 0.77; 95% CI, 0.65–0.93) (**Fig 3**). Results of the full multivariable model are shown in the **S1 Fig in S1 File**. The estimated 90-day mortality was 41.3% (95% CI, 38.9–43.8%) in the early initiation group and 51.3% (95% CI, 46.1–56.5%) in the late initiation group (risk difference, –9.9% [95% CI, –15.9 to –4.0%]).

### Sensitivity analyses

Results were similar across all three sensitivity analyses. Specifically, patients who initiated IMV early had lower mortality as compared to those who initiated IMV late in the analysis in which discharged patients were kept in the risk set until day 90 (HR 0.80 [95% CI, 0.66–0.95]), in the analysis in which patients who died in the first 7 days of ICU admission were excluded (HR 0.66 [95% CI, 0.53–0.81]), and in the analysis that accounted for ICU admission date, hospital geographic region, and hospital type (HR 0.75 [95% CI, 0.62–0.90]) (**Fig 3**).

### Exploratory analysis

We also conducted an exploratory analysis in which patients not intubated on ICU days 1–7 were included in the late IMV group (**S2 Fig in S1 File**). Those in the early IMV group were generally sicker those in the late IMV group, including being considerably more likely to have shock on ICU admission (243/1460 [16.6%] vs. 8/917 [0.9%]). Other baseline characteristics are shown in **S2 Table in S1 File**. After multivariable adjustment, there was no difference in mortality between those in the early vs. late IMV groups (adjusted HR 1.03 [95% CI, 0.80–1.32]) (**Fig 3**).

### Subgroup analyses

The association between early versus late initiation of IMV and time-to-death was similar across each of the following subgroups: age (P for interaction = 0.61); sex (P for interaction = 0.80); race (P for interaction = 0.55); BMI (P for interaction = 0.47); chronic lung disease (P for interaction = 0.27); time from symptom onset to ICU admission (P for interaction = 0.88); inflammation (P for interaction = 0.18); corticosteroid use (P for interaction = 0.16); and number of ICU beds (P for interaction = 0.63) (**Fig 3**).

## Discussion

In this multicenter cohort study of 1879 critically ill adults with COVID-19 admitted to ICUs across the United States, patients who initiated IMV in the first 2 days of ICU admission had a 23% lower adjusted risk of death compared with those who initiated IMV on ICU days 3–7. Results were similar in sensitivity and subgroup analyses. However, in an exploratory analysis in which patients not intubated on ICU days 1–7 were included in the late IMV group, we found no difference in mortality between groups.

Several studies have investigated the association between timing of IMV initiation and death in critically ill patients with COVID-19. A meta-analysis published in 2021 included 8944 critically ill patients with COVID-19 from 12 observational studies, and concluded that timing of intubation has no effect on mortality [12]. However, there was no upper limit on the time to intubation in the “late” initiation group, which likely resulted in the inclusion of patients with heterogeneous clinical features and trajectories. Moreover, 6469 of the 8944 patients (72%) were derived from only two studies [23, 24], one of which lacked key data on severity-of-illness parameters and treatments received [24], and both of which lacked these data at the time of IMV initiation. Since these studies did not account for illness severity at the time of IMV initiation, they were unable to account for worsening clinical trajectory, an important potential confounder, as we did in our analysis. Several studies published more recently found that delayed intubation in critically ill patients with COVID-19 [15, 25], or an initial approach using noninvasive positive-pressure ventilation [26], are associated with increased mortality, consistent with observations in non-COVID-19-ARDS [14, 27, 28].

As practitioners during these particularly challenging early pandemic times, the paucity of high-quality data to guide management decisions led to wide variation in clinical practice [1, 29], which led to “pseudo-randomization” of patients to various therapies and therapeutic strategies. We previously leveraged this natural experiment and the detailed data collected in STOP-COVID to successfully predict the outcomes of subsequent phase 3 randomized clinical trials (RCTs) [4, 30], including the finding that early treatment with tocilizumab [31] has a mortality benefit whereas therapeutic-dose anticoagulation [32] does not in critically ill patients with COVID-19. As with the above therapies, it is likely that there was similarly wide variation in intubation practices as well. Some centers routinely intubated COVID-19 patients early in their disease course based on: 1) initial reports of rapid deterioration of seemingly stable COVID-19 patients; 2) concerns that the time required to don personal protective equipment might preclude later more emergent intubations; and 3) hypothesized risk of P-SILI from the large tidal volumes and transpulmonary pressures observed among spontaneously breathing patients early in the course of respiratory failure. Our findings in the current study are consistent with the hypothesis that early intubation improves outcomes by allowing earlier implementation of lung protective ventilation and avoidance of a deleterious period of spontaneous respiration.

In a post-hoc exploratory analysis, we found no association with mortality when patients not intubated on ICU days 1–7 were included in the late IMV group. In an actual RCT of early vs. late initiation of IMV, some patients assigned to the late IMV group would have improvement in their clinical status and would therefore never be intubated. Accordingly, including such patients in the late IMV group in the current analyses would emulate an RCT more closely, at least in theory [33]. However, by including those not intubated on ICU days 1–7 in the late IMV group, we found considerable differences in the baseline characteristics of the early vs. late IMV groups, such as a nearly 20-fold higher prevalence of shock on ICU admission in the early compared to late group, along with higher renal- and coagulation-SOFA scores (**S2 Table in S1 File**). Although we used multivariable models in an attempt to adjust for these differences, it is likely that residual confounding persisted given the severe imbalance to being with. The discordant findings between our primary vs. exploratory analyses highlight some of the key challenges in using observational data to address the question of early vs. late initiation of IMV, particularly in the handling of those patients who were never intubated.

Our study has several strengths. First, in contrast to the studies included in the meta-analysis described above, we designed our inclusion and exclusion criteria to be representative of those that would be used in a hypothetical RCT of early versus delayed initiation of IMV, excluding patients who would be ineligible to participate in such a trial (e.g., those with severe hypoxemia [PaO_2_:FiO_2_ ratio <100 mm Hg] or refractory shock on ICU admission). Second, we used analytic approaches to prevent immortal time bias and to comprehensively adjust for confounding, leveraging the detailed and longitudinal severity-of-illness data collected in STOP-COVID. Third, whereas most prior studies were single-center, ours included data from multiple geographically-diverse hospitals across the United States, thereby increasing the generalizability of our findings. Fourth, our results were consistent across multiple sensitivity and subgroup analyses.

We also acknowledge several limitations. First, as with all observational analyses, we cannot exclude the possibility of residual confounding, and our findings should therefore be interpreted cautiously. Specifically, despite the comprehensive multivariable adjustment for severity-of-illness, it is possible that improved outcomes were seen here from early intubation of less sick patients. Second, we did not have detailed data on oxygen delivery modality prior to initiation of IMV, nor data on ventilator settings or lung compliance during IMV. Third, our dataset did not specify whether patients receiving IMV on ICU day 1 were intubated prior to or after ICU admission. In an actual RCT of early versus delayed IMV, patients intubated prior to ICU admission would be excluded, and therefore inclusion of such patients in the current analyses could have resulted in confounding and selection bias [34]. However, the bias would have been in the direction of finding early IMV to be harmful, as such patients are likely to have been sicker than those intubated later. Fourth, in an attempt to limit patient heterogeneity, we did not include non-intubated patients in our primary analyses, though in an actual RCT of early versus delayed IMV there would inevitably be patients in the delayed group who would not ultimately receive IMV. Fifth, center-related factors (e.g., adequacy of resources and local policies) and clinician-related factors (e.g., background and training) could also have affected the timing of IMV [35, 36], and may not have been fully accounted for in our models. However, our models were adjusted for hospital size (number of ICU beds), and in sensitivity analyses that further accounted for hospital geographic location, hospital type, and date of ICU admission, we found consistent findings as our primary analysis. Finally, data were obtained from patients admitted early on in the pandemic. Accordingly, the generalizability of these findings to patients today may be limited given the emergence of vaccines, novel therapies and variants, and potential differences in thresholds for intubation early on in the pandemic versus today.

Additionally, we defined early and late initiation of IMV according to its timing in relation to ICU admission, since we did not have detailed data on the timing of acute hypoxemic respiratory failure onset prior to ICU admission. We excluded patients who were transferred from other hospitals, as well as those hospitalized for more than 1 week prior to ICU admission, to minimize misclassification of patients into the early and late IMV groups. However, we acknowledge that some studies have defined early and late initiation of IMV using alternative parameters, such as the amount of oxygen support at the time of intubation. Along these lines, Yamamoto and colleagues recently defined early intubation as receipt of ≤6 L/min of oxygen, and found that patients with COVID-19 intubated “early” had decreased in-hospital mortality compared to those intubated “late” [37]. A notable disadvantage of this approach, compared to ours, is the strong possibility of residual confounding, since those in the early group had, by definition, less severe hypoxemia compared to those in the late group.

## Conclusion

In summary, we found that patients who initiated IMV early had a considerably lower adjusted risk of death compared to those who initiated IMV late. Future studies, ideally in the form of carefully designed and adequately powered RCTs, are needed to identify optimal therapeutic strategies surrounding IMV in critically ill patients with COVID-19 to reduce the very high mortality in this population.

## Supporting information

S1 File (DOCX)
