# Supplementary material for: Timing of invasive mechanical ventilation and death in critically ill adults with COVID-19: A multicenter cohort study
Source: PLoS One. 2023 Jun 28;18(6):e0285748. doi: 10.1371/journal.pone.0285748 (PMC10306211; doi:10.1371/journal.pone.0285748)
Supplement: S1 File — (DOCX) [file pone.0285748.s001.docx]

**supplemental material**

**Timing of invasive mechanical ventilation and death in critically ill adults with COVID-19: A Multicenter Cohort Study**

| **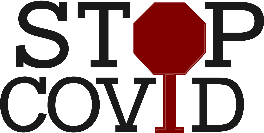** | **Study of the Treatment and Outcomes in Critically Ill Patients with COVID-19** |
| --- | --- |

**TABLE OF CONTENTS**

**STOP-COVID Investigators…………………………………………………………………………………….3–6**

**Supplemental Methods………………………………..…………………………………………………………..7**

**S1 Fig. Multivariable-Adjusted Risk Model for Death………………………………………………………..8**

**S2 Fig. Study cohort for Exploratory Analysis…………………………………………...............................9**

**S1 Table. Causes of Death………………………………………………………………………………………10**

**S2 Table. Baseline Characteristics of Patients in Exploratory Analysis……………………………11-12**

**Supplemental References……………………………………………………………………………………….13**

**STOP-COVID INVESTIGATORS**

**Baylor College of Medicine:** Carl P. Walther*, Samaya J. Anumudu

**Baylor University Medical Center:** Justin Arunthamakun*, Kathleen F. Kopecky, Gregory P. Milligan, Peter A. McCullough, Thuy-Duyen Nguyen

**Beth Israel Deaconess Medical Center:** Shahzad Shaefi*, Megan L. Krajewski, Sidharth Shankar, Ameeka Pannu, Juan D. Valencia

**Boston Medical Center:** Sushrut S. Waikar*, Zoe A. Kibbelaar

**Cook County Health:** Ambarish M. Athavale*, Peter Hart, Shristi Upadhyay, Ishaan Vohra

**Cooper University Health Care:** Adam Green*, Jean-Sebastien Rachoin, Christa A. Schorr, Lisa Shea

**Duke University Medical Center:** Daniel L. Edmonston*, Christopher L. Mosher

**Hackensack Meridian Health Mountainside Medical Center:** Alexandre M. Shehata*, Zaza Cohen, Valerie Allusson, Gabriela Bambrick-Santoyo, Noor ul aain Bhatti, Bijal Mehta, Aquino Williams

**Hackensack Meridian Health Hackensack University Medical Center:** Samantha K. Brenner*, Patricia Walters, Ronaldo C. Go, Keith M. Rose

**Harvard T.H. Chan School of Public Health:** Miguel A. Hernán

**Harvard University:** Rebecca Lisk, Amy M. Zhou, Ethan C. Kim

**Icahn School of Medicine at Mount Sinai:** Lili Chan*, Kusum S. Mathews*, Steven G. Coca, Deena R. Altman, Aparna Saha, Howard Soh, Huei Hsun Wen, Sonali Bose, Emily A. Leven, Jing G. Wang, Gohar Mosoyan, Girish N. Nadkarni, Pattharawin Pattharanitima, Emily J. Gallagher

**Indiana University School of Medicine/Indiana University Health:** Allon N. Friedman*, John Guirguis, Rajat Kapoor, Christopher Meshberger, Katherine J. Kelly

**Johns Hopkins Hospital:** Chirag R. Parikh*, Brian T. Garibaldi, Celia P. Corona-Villalobos, Yumeng Wen, Steven Menez, Rubab F. Malik, Elena Cervantes, Samir Gautam

**Kings County Hospital Center**: Mary C. Mallappallil*, Jie Ouyang, Sabu John, Ernie Yap, Yohannes Melaku, Ibrahim Mohamed, Siddartha Bajracharya, Isha Puri, Mariah Thaxton, Jyotsna Bhattacharya, John Wagner, Leon Boudourakis

**Loma Linda University:** H. Bryant Nguyen*, Afshin Ahoubim

**Mass General Brigham:** Brigham and Women’s Hospital, Brigham and Women’s Faulkner Hospital, Massachusetts General Hospital, and Newton Wellesley Hospital - David E. Leaf*, Shruti Gupta*, Meghan E. Sise, Erik T. Newman, Samah Abu Omar, Kapil K. Pokharel, Shreyak Sharma, Harkarandeep Singh, Simon Correa, Tanveer Shaukat, Omer Kamal, Wei Wang, Heather Yang, Jeffery O. Boateng, Meghan Lee, Ian A. Strohbehn, Jiahua Li, Ariel L. Mueller

**Mayo Clinic, Arizona:** Leslie F. Thomas*, Dheeraj Reddy Sirganagari

**Mayo Clinic, Florida:** Pramod K. Guru*

**Mayo Clinic, Rochester:** Kianoush Kashani*, Shahrzad Tehranian

**Medical College of Wisconsin:** Yan Zhou,* Paul A. Bergl, Jesus Rodriguez, Jatan A. Shah, Mrigank S. Gupta

**MedStar Georgetown University Hospital:** Princy N. Kumar*, Deepa G. Lazarous, Seble G. Kassaye

**Montefiore Medical Center/Albert Einstein College of Medicine**: Michal L. Melamed*, Tanya S. Johns. Ryan Mocerino, Kalyan Prudhvi, Denzel Zhu, Rebecca V. Levy, Yorg Azzi, Molly Fisher, Milagros Yunes, Kaltrina Sedaliu, Ladan Golestaneh, Maureen Brogan, Neelja Kumar, Michael Chang, Jyotsana Thakkar

**New York-Presbyterian Queens Hospital**: Ritesh Raichoudhury*, Akshay Athreya, Mohamed Farag

**New York-Presbyterian/Weill Cornell Medical Center:** Edward J. Schenck*, Soo Jung Cho, Maria Plataki, Sergio L. Alvarez-Mulett, Luis G. Gomez-Escobar, Di Pan, Stefi Lee, Jamuna Krishnan, William Whalen

**New York University Langone Hospital:** David Charytan*, Ashley Macina, Sobaata Chaudhry, Benjamin Wu, Frank Modersitzki

**Northwestern Memorial Hospital:** Northwestern University Feinberg School of Medicine - Anand Srivastava*, Alexander S. Leidner, Carlos Martinez, Jacqueline M. Kruser, Richard G. Wunderink, Alexander J. Hodakowski

**Ochsner Medical Center:** Juan Carlos Q. Velez*, Eboni G. Price-Haywood, Luis A. Matute-Trochez, Anna E. Hasty, Muner MB. Mohamed

**Oregon Health and Science University Hospital:** Rupali S. Avasare*, David Zonies*

**ProMedica Health System**: Roberta Redfern,* Nicholas S. Cairl, Gabriel Naimy, Abeer Abu-Saif, Danyell Hall, Laura Bickley

**Renown Health:** Chris Rowan*, Farah Madhani-Lovely*

**Rush University Medical Center:** Vasil Peev*, Jochen Reiser, John J. Byun, Andrew Vissing, Esha M. Kapania, Zoe Post, Nilam P. Patel, Joy-Marie Hermes

**Rutgers/New Jersey Medical School:** Anne K. Sutherland*, Amee Patrawalla, Diana G. Finkel, Barbara A. Danek, Sowminya Arikapudi, Jeffrey M. Paer, Peter Cangialosi, Mark Liotta

**Rutgers/Robert Wood Johnson Medical School:** Jared Radbel*, Jag Sunderram, Sonika Puri, Jayanth S. Vatson, Matthew T. Scharf, Ayesha Ahmed, Ilya Berim,

**Stanford Healthcare:** Stanford University School of Medicine – Shuchi Anand*, Joseph E. Levitt, Pablo Garcia

**Temple University Hospital:** Suzanne M. Boyle*, Rui Song, Ali Arif

**Thomas Jefferson Health**: Jingjing Zhang*, Sang Hoon Woo, Xiaoying Deng, Goni Katz-Greenberg, Katharine Senter

**Tulane Medical Center:** Moh’d A. Sharshir*, Vadym V. Rusnak

**United Health Services Hospitals:** Muhammad Imran Ali, Terri Peters, Kathy Hughes

**University of Colorado Anschutz Medical Campus:** Anip Bansal*, Amber S. Podoll, Michel Chonchol, Sunita Sharma, Ellen L. Burnham

**University Hospitals Cleveland Medical Center:** Arash Rashidi*, Rana Hejal

**University of Alabama-Birmingham Hospital:** Eric Judd*, Laura Latta, Ashita Tolwani

**University of California-Davis Medical Center:** Timothy E. Albertson*, Jason Y. Adams

**University of California-Los Angeles Medical Center:** Ronald Reagan-UCLA Medical Center - Steven Y. Chang*, Rebecca M. Beutler; Santa Monica-UCLA Medical Center – Carl E. Schulze

**University of California-San Diego Medical Center:** Etienne Macedo*, Harin Rhee

**University of California-San Francisco Medical Center:** Kathleen D. Liu*, Vasantha K. Jotwani

**University of Chicago Medical Center:** Jay L. Koyner*

**University of Florida Health-Gainesville:** Chintan V. Shah*

**University of Florida-Health-Jacksonville:** Vishal Jaikaransingh*

**University of Illinois Hospital and Health Sciences System:** Stephanie M. Toth-Manikowski*, Min J. Joo*, James P. Lash

**University of Kentucky Medical Center:** Javier A. Neyra*, Nourhan Chaaban

**University Medical Center of Southern Nevada:** Alfredo Iardino, Elizabeth H. Au, Jill H. Sharma

**University of Miami Health System:** Marie Anne Sosa*, Sabrina Taldone, Gabriel Contreras, David De La Zerda, Alessia Fornoni, Hayley B. Gershengorn

**University of Michigan:** Salim S. Hayek*, Pennelope Blakely, Hanna Berlin, Tariq U. Azam, Husam Shadid, Michael Pan, Patrick O’ Hayer, Chelsea Meloche, Rafey Feroze, Kishan J. Padalia, Abbas Bitar, Jeff Leya, John P. Donnelly, Andrew J. Admon

**University of North Carolina School of Medicine:** Jennifer E. Flythe*, Matthew J. Tugman, Emily H. Chang

**University of Oklahoma Health Sciences Center:** Brent R. Brown*

**University of Pennsylvania Health System:** Amanda K. Leonberg-Yoo*, Ryan C. Spiardi, Todd A. Miano, Meaghan S. Roche, Charles R. Vasquez

**University of Pittsburgh Medical Center:** Amar D. Bansal*, Natalie C. Ernecoff, Sanjana Kapoor, Siddharth Verma, Huiwen Chen

**University of Tennessee Health Science Center and Memphis VA Medical Center/Methodist University Hospital** – Csaba P. Kovesdy*, Miklos Z. Molnar*, Ambreen Azhar

**University of Texas Southwestern Medical Center and Parkland Health and Hospital System:** S. Susan Hedayati*, Mridula V. Nadamuni, Shani Shastri, Duwayne L. Willett

**University of Vermont Larner College of Medicine:** Samuel A.P. Short

**University of Virginia Health System:** Amanda D. Renaghan*, Kyle B. Enfield

**University of Washington Medical Center:** Pavan K. Bhatraju*, A. Bilal Malik

**Vanderbilt University Medical Center:** Matthew W. Semler

**Washington University in St. Louis/Barnes Jewish Hospital**: Anitha Vijayan*, Christina Mariyam Joy, Tingting Li, Seth Goldberg, Patricia F. Kao

**Wellforce Health System:** Lowell General Hospital **-** Greg L. Schumaker*, Tufts Medical Center - Nitender Goyal*, Anthony J. Faugno, Greg L. Schumaker, Caroline M. Hsu, Asma Tariq, Leah Meyer, Ravi K. Kshirsagar, Daniel E. Weiner

**Westchester Medical Center:** Marta Christov*, Jennifer Griffiths, Sanjeev Gupta, Aromma Kapoor

**Yale School of Medicine:** Perry Wilson,* Tanima Arora, Ugochukwu Ugwuowo

*Site Principal Investigator

**Supplemental Methods**

**Data Collection and Validation**

Data were collected using REDCap, a secure, HIPAA-compliant, web-based application. Wherever possible, data were captured using checkboxes rather manual entry to minimize keystroke errors. For data that required keystroke entry (e.g., laboratory values), we implemented validation ranges to flag potential errors in real-time. We also implemented automated data validation rules to flag errors in dates (e.g., if the date of death was entered as being before the date of ICU admission). In addition, all data were manually reviewed, and values that appeared incongruent or out of range were manually validated by confirming the accuracy of the data with the collaborator who entered it.

**Covariates included in Multivariable Cox Model**

A. Baseline covariates

1. Age: 18-49 (REF); 50-59; 60-69; ≥70

2. Male vs. female (REF)

3. Race: White (REF) vs. non-White/Other/Unknown

4. Body mass index (kg/m^2^): <25 (REF); 25-29; 30-34; 35-39.9; ≥40

5. Hypertension

6. Diabetes mellitus

7. Coronary artery disease

8. Congestive heart failure

9. Chronic lung disease (composite of COPD or current or former smoker)

10. Active malignancy

11. Time from symptom onset to ICU admission (days): 0 to 3; >3 (REF)

12. Hospital size (#pre-COVID ICU beds): ≥100 (REF); 50-99; <50

B. Severity-of-illness covariates assessed on the day of IMV initiation

1. Renal, liver, and coagulation components of the Sequential Organ Failure Assessment (SOFA) score (1):

|  |  | **Categories** | | | |
| --- | --- | --- | --- | --- | --- |
|  | **0** | **1** | **2** | **3** | **4** |
| SOFA Renal (Cr, UOP, and acute RRT) | Cr<1.2 and UOP≥500 | Cr 1.2–1.9 and UOP≥500 | Cr 2.0–3.4 and UOP≥500 | Cr 3.5–4.9 or UOP<500 | Cr ≥5 or UOP<200 or acute RRT or ESRD |
| SOFA Liver (Bilirubin) | <1.2 | 1.2–1.9 | ≥2* | --- | --- |
| SOFA Coagulation (Platelets) | ≥150 | 100–149 | ≤99* | --- | --- |

Abbreviations: Cr, creatinine (mg/dl); ESRD, end stage renal disease; RRT, renal replacement therapy; UOP, urine output. *The liver and coagulation components of the SOFA score were binned due to low frequency of events in categories “3” and “4”.

2. PaO_2_:FiO_2_ ratio on the day of IMV initiation: ≥300 (REF); 200-299; <200

3. Shock on the day of IMV initiation, defined as receipt of ≥2 vasopressors

4. Treatments received on the day of IMV initiation or prior: corticosteroids; tocilizumab; prone positioning; neuromuscular blockade

5. Inflammation. Three mutually exclusive categories were created: inflamed, non-inflamed, or missing. Inflamed was defined as at least one of the following on the day of IMV initiation or prior: C-reactive protein >100 mg/L, interleukin-6 >80 pg/ml, or ferritin >1,000 ng/mL. Non-inflamed was defined as at least one value that was below the threshold and no value that was above the threshold for the above parameters. Missing was defined as all three values being missing. The above thresholds were chosen based on prior studies (2-4).

**S1 Fig. Multivariable-Adjusted Risk Model for Death.** Abbreviations: IMV, invasive mechanical ventilation; REF, reference group; SOFA, Sequential Organ Failure Assessment.

**
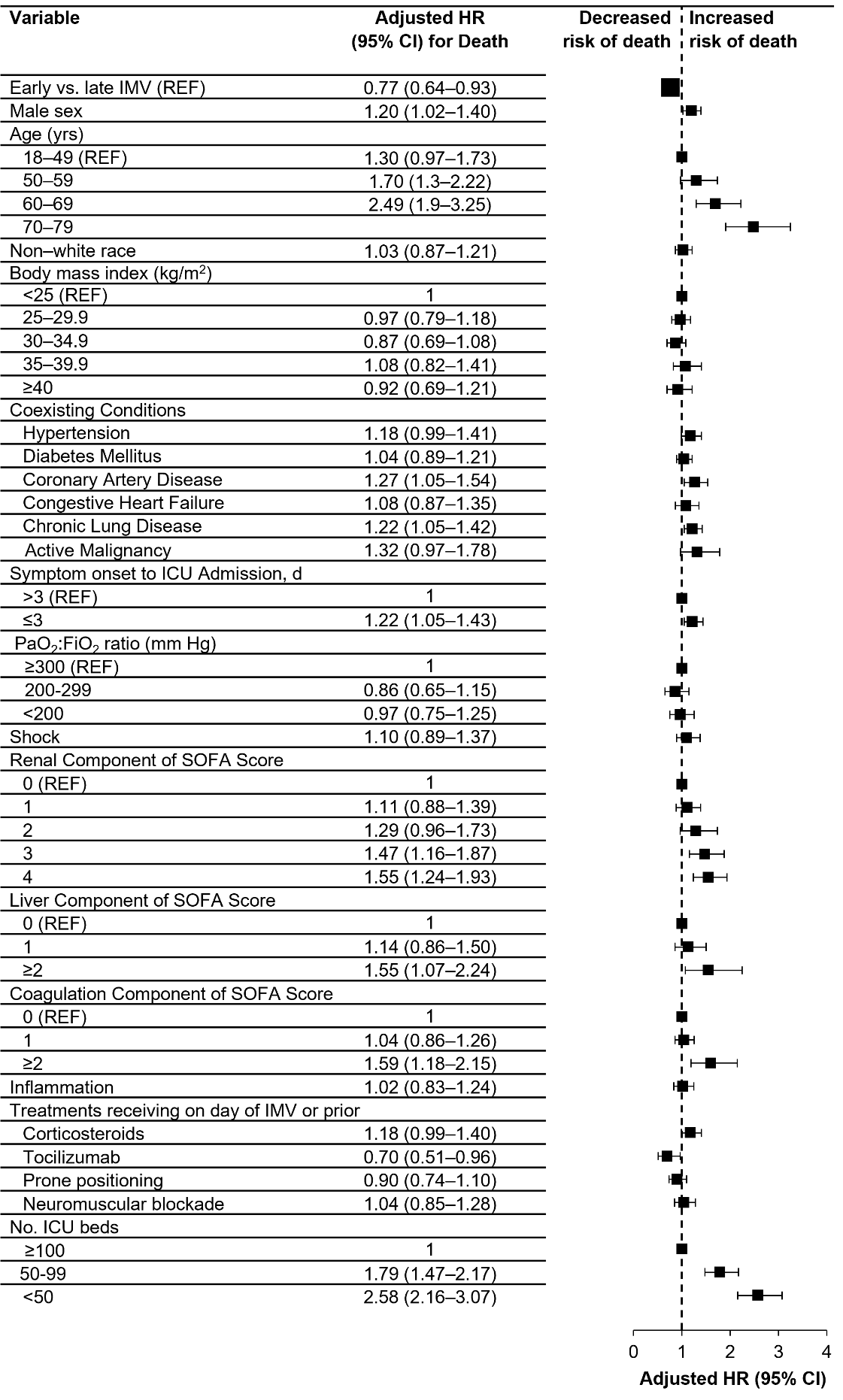
**

**S2 Fig. Study cohort for Exploratory Analysis.** In this analysis, patients not intubated on ICU days 1-7 were included in the late IMV group. Abbreviations: IMV, invasive mechanical ventilation; PaO2:FiO2, ratio of the partial pressure of arterial oxygen over the fraction of inspired oxygen; O_2_, oxygen. ^a^Defined as no supplemental O_2_ or only minimal supplemental O_2_ receipt (nasal cannula or simple face mask).

**
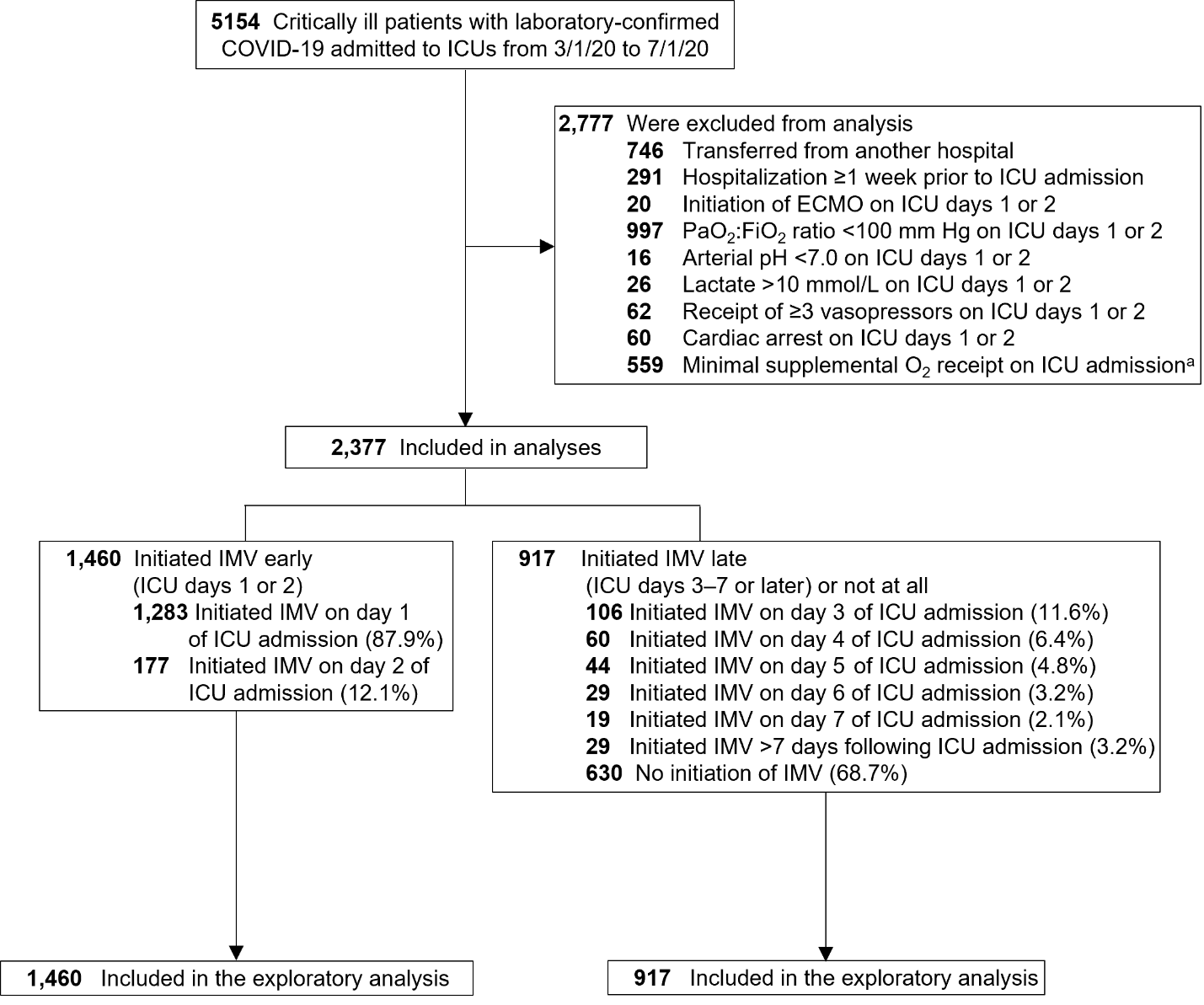
**

**S1 Table. Causes of Death**

| **Cause of Death – no. (%)** | **Early initiation of IMV**  **(N=644)** | **Late initiation of IMV (N=180)** |
| --- | --- | --- |
| ARDS/respiratory failure | 576 (89.4) | 167 (92.8) |
| Congestive heart failure | 55 (8.5) | 15 (8.3) |
| Septic shock | 251 (29) | 80 (44.4) |
| Acute kidney injury | 228 (35.4) | 67 (37.2) |
| Liver failure | 23 (3.6) | 12 (6.7) |
| Other causes | 100 (15.5) | 24 (13.3) |

Note: Patients could have had more than 1 cause of death, and thus percentages sum to more than 100.

Abbreviations: ARDS, acute respiratory distress syndrome; IMV, invasive mechanical ventilation

**S2 Table. Baseline Characteristics of Patients in Exploratory Analysis**

|  | **Treatment group** | |  |
| --- | --- | --- | --- |
| **Characteristic** | **Early initiation of IMV**  **(n = 1460)** | **Late or no initiation of IMV**  **(n = 917)** | **P Value** |
| **Demographics** |  |  |  |
| Age – median (IQR) | 63 (54─72) | 62 (52─73) | 0.34 |
| Age – no. (%) |  |  |  |
| 18–49 | 266 (18.2) | 197 (21.5) | 0.07 |
| 50–59 | 296 (20.3) | 190 (20.7) |  |
| 60–69 | 448 (30.7) | 240 (26.2) |  |
| ≥70 | 450 (30.8) | 290 (31.6) |  |
| Male sex – no. (%) | 938 (64.2) | 537 (58.6) | 0.005 |
| Race – no. (%) |  |  |  |
| White | 583 (40.0) | 379 (41.4) | 0.48 |
| Non-White/Other/Unknown | 876 (60.0) | 536 (58.6) |  |
| BMI (kg/m^2^) – median (IQR) | 29.9 (26.1─34.5) | 30.4 (26.5─36.7) | 0.03 |
| **Coexisting conditions – no. (%)** |  |  |  |
| Hypertension | 904 (61.9) | 542 (59.1) | 0.17 |
| Diabetes mellitus | 630 (43.2) | 376 (41.0) | 0.30 |
| Coronary artery disease | 180 (12.3) | 134 (14.6) | 0.11 |
| Congestive heart failure | 126 (8.6) | 116 (12.6) | 0.002 |
| Chronic lung disease | 452 (31.0) | 324 (35.3) | 0.03 |
| Active malignancy | 60 (4.1) | 38 (4.1) | 0.97 |
| **Days from symptom onset to ICU admission – no. (%)** | |  |  |
| ≤3 | 331 (22.7) | 202 (22.1) | 0.70 |
| >3 | 1124 (77.3) | 713 (77.9) |  |
| **Severity-of-illness – no. (%)^a^** | |  |  |
| PaO_2_:FiO_2_, mm Hg^b^ |  |  |  |
| ≥300 | 171 (12.9) | 4 (7.7) | 0.34 |
| 200-299 | 358 (26.9) | 12 (23.1) |  |
| <200 | 800 (60.2) | 36 (69.2) |  |
| Shock^c^ | 243 (16.6) | 8 (0.9) | <0.001 |
| Renal SOFA score^d^ |  |  |  |
| 0 | 799 (54.7) | 732 (79.8) | <0.001 |
| 1 | 212 (14.5) | 55 (6.0) |  |
| 2 | 78 (5.3) | 19 (2.1) |  |
| 3 | 218 (14.9) | 78 (8.5) |  |
| 4 | 153 (10.5) | 33 (3.6) |  |
| Liver SOFA score^e^ |  |  |  |
| 0 | 1315 (90.1) | 850 (92.7) | 0.08 |
| 1 | 109 (7.5) | 53 (5.8) |  |
| 2–4 | 36 (2.5) | 14 (1.5) |  |
| Coagulation SOFA score^f^ |  |  |  |
| 0 | 1147 (78.6) | 758 (82.7) | 0.03 |
| 1 | 236 (16.2) | 127 (13.8) |  |
| 2–4 | 77 (5.3) | 32 (3.5) |  |
| Inflammation^g^ |  |  |  |
| Inflamed (≥1 elevated marker) | 1034 (81.4) | 567 (74.0) | <0.001 |
| Non-inflamed (no elevated markers) | 237 (18.6) | 199 (26.0) |  |
| **Treatments – no. (%)^h^** | |  |  |
| Corticosteroids | 200 (13.7) | 125 (13.6) | 0.96 |
| Tocilizumab | 70 (4.8) | 71 (7.7) | 0.003 |
| Prone positioning | 194 (13.3) | 159 (17.3) | 0.007 |
| Neuromuscular blockade | 172 (11.8) | 17 (1.9) | <0.001 |
| **Number of ICU beds – no. (%)** |  |  |  |
| <50 | 519 (35.5) | 269 (29.3) | 0.003 |
| 50-99 | 418 (28.6) | 267 (29.1) |  |
| ≥100 | 523 (35.8) | 381 (41.5) |  |

**S2 Table legend**

^a^Severity-of-illness characteristics were assessed on ICU admission.

^b^PaO_2_:FiO_2_ was only assessed in patients receiving IMV. If multiple PaO_2_ values were available on the same day, the lowest value was recorded, along with the corresponding FiO_2_.

^c^Defined as receipt of two or more vasoactive agents, including phenylephrine, epinephrine, norepinephrine, vasopressin, dopamine, dobutamine, and milrinone.

^d^Renal SOFA scores were calculated by considering the daily SCr, the daily UOP, receipt of RRT, and ESRD. Category 0, SCr <1.2 mg/dl and UOP ≥500 ml; category 1, SCr 1.2–1.9 mg/dl and UOP ≥500 ml; category 2, SCr 2.0 – 3.4 mg/dl and UOP ≥500 ml; category 3, SCr 3.5–4.9 mg/dl or UOP <500 ml; category 4, SCr ≥5 mg/dl, UOP <200 ml, receipt of RRT, or ESRD. Higher scores indicate more severe renal dysfunction.

^e^Liver SOFA scores were calculated by determining the daily bilirubin level. Category 0, bilirubin <1.2 mg/dl; category 1, bilirubin 1.2–1.9 mg/dl; category 2–4, bilirubin ≥2 mg/dl. Higher scores indicate more severe liver dysfunction. Categories 2, 3, and 4 were binned due to a low frequency of events in categories 3 and 4.

^f^Coagulation SOFA scores were calculated by determining the daily platelet level (per mm^3^). Category 0, platelet count ≥150; category 1, platelet count 100–149; category 2–4, platelet count <100. Higher scores indicate more severe dysfunction of the coagulation system. Categories 2, 3, and 4 were binned due to a low frequency of events in categories 3 and 4.

^g^Inflamation was defined as at least one of the following on the day of IMV initiation or prior: C-reactive protein >100 mg/L, interleukin-6 >80 pg/ml, or ferritin >1,000 ng/mL. Non-inflamed was defined as at least one value below the thresholds above, and no values that were above the thresholds. These thresholds above were selected based on prior studies (2-4).

^h^Refers to treatments received on the day of ICU admission.

Abbreviations: BMI, body mass index; ESRD, end stage renal disease; ICU, intensive care unit; IMV, invasive mechanical ventilation; PaO_2_:FiO_2_, ratio of the partial pressure of arterial oxygen over the fraction of inspired oxygen; RRT, renal replacement therapy; SCr, serum creatinine; SOFA, Sequential Organ Failure Assessment; UOP, urine output.

Data regarding BMI were missing for 51 patients (3.5%) in the early IMV group and for 33 patients (3.6%) in the late or no IMV group.

Data regarding days from symptom onset to ICU admission were missing for 5 patients (0.3%) in the early IMV group and for 2 patients (0.2%) in the late or no IMV group.

Data regarding PaO_2_:FiO_2_ were missing for 131 patients (9%) in the early IMV group and for 865 patients (94.3%) in the late or no IMV group.

Data regarding inflammation were missing for 189 patients (12.9%) in the early IMV group and for 151 patients (16.5%) in the late or no IMV group.

All other data are complete.

**Supplemental References**

1. Vincent JL, Moreno R, Takala J, et al. The SOFA (Sepsis-related Organ Failure Assessment) score to describe organ dysfunction/failure. On behalf of the Working Group on Sepsis-Related Problems of the European Society of Intensive Care Medicine. *Intensive Care Med* 1996;22(7):707-710.

2. Herold T, Jurinovic V, Arnreich C, et al. Elevated levels of IL-6 and CRP predict the need for mechanical ventilation in COVID-19. *J Allergy Clin Immunol* 2020.

3. Landry A, Docherty P, Ouellette S, et al. Causes and outcomes of markedly elevated C-reactive protein levels. *Can Fam Physician* 2017;63(6):e316-e323.

4. Moore C, Jr., Ormseth M, Fuchs H. Causes and significance of markedly elevated serum ferritin levels in an academic medical center. *J Clin Rheumatol* 2013;19(6):324-328.
